# Caffeine Acts as an Agonist of Siglec-6, Inhibits MRGPRX2-Triggered Mast Cell Degranulation and Anaphylactoid Reactions

**DOI:** 10.1155/mi/9580121

**Published:** 2025-08-30

**Authors:** Yuanyuan Ding, Na Wang, Chenrui Zhao, Hongfen Du, Yujuan Yuan, Tao Zhang, Hongli An

**Affiliations:** ^1^Center for Translational Medicine, The First Affiliated Hospital of Xi'an Jiaotong University, Xi'an 710061, China; ^2^College of Pharmacy, Health Science Center, Xi'an Jiaotong University, Xi'an 710061, Shaanxi, China; ^3^Department of Otolaryngology, Affiliated Hospital of North China University of Science and Technology, Tangshan 063000, China; ^4^Key Laboratory for Tumor Precision Medicine of Shaanxi Province, The First Affiliated Hospital of Xi'an Jiaotong University, Xi'an 710061, Shaanxi, China

**Keywords:** anaphylactoid reaction, caffeine, mast cell degranulation, MRGPRX2, Siglec-6

## Abstract

**Background:** Mast cells (MCs) are effectors of anaphylactoid reactions. Mas-related G-protein-coupled receptor X2 (MRGPRX2) receptor mediates the direct activation of MCs in anaphylactoid disease. Siglec-6 negatively regulates MC activation and is a promising target in the development of antianaphylactoid reaction drugs. While caffeine exhibits an inhibitory effect against anaphylactic shock, the molecular mechanisms underlying these activities remain unknown.

**Objectives:** Our objective was to investigate the inhibitory effect of caffeine and its underlying molecular mechanism in MRPGRX2-induced MC activation and anaphylactoid reactions.

**Methods:** Local and systemic anaphylactoid reactions in mice and in vitro MC activation experiments were conducted to investigate the effects of caffeine on anaphylactoid reactions. Molecular docking and surface plasmon resonance (SPR) experiments were used to predict and verify the molecular target of caffeine activity. siRNA silencing and western blot analyses were utilized to investigate the molecular mechanisms underlying caffeine activity.

**Results:** Caffeine inhibited local and systemic anaphylactoid reactions in mice and attenuated MRGPRX2-induced MC activation. Release of β-hexosaminidase, histamine, and Ca^2+^ in siRNA-Siglec-6-laboratory allergic disease 2 (LAD2) cells was significantly higher than in NC-LAD2 cells. The binding affinity between caffeine and Siglec-6 protein is with a calculated *K*_D_ of 1.76 × 10^−7^ mol/L. Caffeine increased Siglec-6 expression, phosphorylation of SHP-1, and dephosphorylation of PLC-γ1, IP3R, and ERK1/2 in the MRGPRX2 signaling pathway. Western blot demonstrated that phosphorylated SHP-1 (p-SHP-1) protein levels showed no increase, and MRGPRX2, phosphorylated PLCγ1 (p-PLCγ1), and phosphorylated ERK1/2 (p-ERK1/2) were abolished with caffeine treatment in Siglec-6-knockdown cells than in NC-knockdown cells. Caffeine suppressed the m-3M3FBS-induced upregulation of p-PLCγ1 and p-ERK1/2 levels.

**Conclusions:** We have demonstrated that caffeine is an agonist of Siglec-6 and that subsequent activation of the ITIM motif of Siglec-6 phosphorylates SHP-1. This arrests MRGPRX2/PLC-γ1/IP3R signal transduction, thereby attenuating anaphylactoid reactions, including anaphylactic shock.

## 1. Introduction

The term “anaphylactoid reaction” is used to describe an IgE-independent allergic-like reaction to a chemical substance, usually an exogenous ligand (e.g., C48/80) or endogenous ligand (e.g., substance P [SP]), that elicits a systemic reaction via activation of mast cells (MCs). Following activation of MCs, various inflammatory and immunomodulatory substances are secreted, including histamine. Interestingly, MCs have recently been identified as a critical factor in several drug adverse reactions [1, 2].

During perioperative care, several nondepolarizing neuromuscular blocking drugs (NMBDs), including atracurium, cis-atracurium, mivacurium, and rocuronium, have been reported to induce drug adverse reactions. For example, mivacurium and atracurium cause skin flushing, erythema, and decreased blood pressure. Importantly, histamine levels have been reported to rise significantly after injection of these NMBDs [3–6]. Rocuronium has been reported to cause especially severe drug adverse reactions, occasionally leading to shock and even death [7].

Most existing therapeutic drugs for the prevention of perioperative anaphylaxis do not meet the diverse clinical needs, and some are not universally applicable. To address patient needs, new small-molecule drugs for the prevention of acute anaphylaxis during the perioperative period must be identified.

Natural compounds are a valuable source for innovative drugs. Caffeine is a well-researched psychoactive alkaloid, and this bioactive compound is commonly consumed worldwide [8]. It possesses anti-inflammatory and antioxidant activities [9, 10], and it exhibits protective effects against cardiovascular disease, cancer, and neurodegenerative diseases, amongst other diseases [11–16]. As a therapeutic drug, caffeine is widely applied to treat apnea in premature infants [17], and it is used in combination with analgesics to relieve pain [18]. In addition, caffeine has been shown to help alleviate allergic diseases, including bronchial asthma and allergic reactions [19, 20]. However, the underlying mechanisms by which caffeine inhibits anaphylactoid reactions remain unclear.

Mas-related G-protein-coupled receptor X2 (MRGPRX2) is widely expressed on human dendritic cells, basophils, and MCs, and it is an important target for effectors of anaphylactoid reactions [21]. Several exogenous ligands (C48/80, ciprofloxacin, etc.) and endogenous ligands (SP, etc.) have been demonstrated to directly activate MRGPRX2 to trigger an anaphylactoid reaction [1]. Clinically, several FDA-approved drugs, including neuromuscular blockers and opioids, have been reported to activate MCs via MRGPRX2 [22, 23]. Siglec receptors are a diverse family of immunoglobulin-like receptors that bind to sialic acid-containing glycans [24, 25]. The members of this family are usually classified as either sequence-conserved receptors or CD33-related, rapidly evolving receptors, and both play a role in innate and adaptive immunity. The cytoplasmic domains of most CD33-related Siglecs have immune receptor tyrosine-based inhibitory motifs (ITIMs) that can recruit tyrosine phosphatases to transmit inhibitory signals through phosphorylation [25]. Siglec-6 is an archetypal CD33-related, rapidly evolving Siglec receptor with high expression in CD34^+^-derived human MCs and the HMC-1 cell line [26]. Siglec-6 activity has been reported to significantly reduce MC activation through MRGPRX2 [27], most likely by regulating intracellular signaling through phosphorylation of SHP-1 and SHP-2 [28]. Hence, Siglec-6 is a potential therapeutic target for anaphylactoid-related diseases. However, research on therapeutic drugs targeting Siglec-6 is insufficient, and additional studies are needed to understand how therapeutic drugs targeting Siglec-6 can negatively regulate the anaphylactoid reaction induced by MRGPRX2.

In our present study, we investigated the antianaphylactoid reaction effects and mechanism of caffeine in vivo and in vitro. An important aim was to investigate whether Siglec-6 plays a role in caffeine-induced inhibition of MC activation and the antianaphylactoid reaction. We also performed experiments to assess whether caffeine is a ligand of Siglec-6, and whether this interaction negatively regulates activation of MCs mediated by MRGPRX2.

## 2. Materials and Methods

### 2.1. Pharmaceuticals and Reagents

Caffeine, C48/80, SP, and Triton X-100 were sourced from Sigma–Aldrich (Merck KGaA). Fluo-3 AM was procured from Thermo Fisher Scientific. Pluronic F-127 gel was acquired from Biotium. StemPro-34 medium and human stem cell factor (SCF) were obtained from Cell Signaling Technology. Evans Blue was procured from Sigma–Aldrich (Merck KGaA). ELISA kits for human and mouse MCP-1, TNF-α, and IL-8 were procured from Sino Biological Inc. The Bb-2000 noninvasive blood pressure analysis system for small Animals was obtained from Beijing Mingxintong Biotechnology Co., Ltd. The FT3403 color LCD economic digital temperature controller was purchased from Guangzhou Taimeike Electronic Technology Co., Ltd.

### 2.2. Cell Lines and Animals

The human MC laboratory allergic disease 2 (LAD2) cell line was acquired from A. Kirshenbaum and D. Metcalfe (NIH, USA). The cells were cultured in StemPro-34 medium supplemented with 10 mL/L StemPro nutritional supplement, penicillin (1:100), streptomycin (1:100), 2 mmol/L glutamine, and 100 ng/mL human SCF. LAD2 cells were maintained at 37°C in a 5% CO_2_ atmosphere.

HEK293 cells expressing MRGPRX2 (MRGPRX2-HEK293) and HEK293 cells expressing empty plasmid (NC-HEK293) were prepared in-house. These cells were cultured in DMEM containing 10% fetal bovine serum, 100 U penicillin, and 100 U streptomycin.

C57BL/6 adult male mice (age, 6–8 weeks; weight, 25 ± 2 g) were purchased from the Experimental Animal Center of Xi'an Jiao-tong University (Xi'an, China). The C57BL/6 mice were anesthetized intraperitoneally with pentobarbital sodium (50 mg/kg).

### 2.3. Ethics Statement

Experimental protocols involving mice were ethically approved by the Biomedical Ethics Committee of Health Science Center of Xi'an Jiaotong University (Date of Approval: May 10, 2023; Certification Number: XJTUAE2023-1621).

### 2.4. Local Anaphylactoid Reactions in C57BL/6 Mice

The C57BL/6 mice were randomly divided into two experimental groups (*n* = 5 per group): a C48/80-induced group and an SP-induced group. Caffeine (1, 2, 4 mg/kg) dissolved in 0.2 mL of physiological saline was administered intraperitoneally to all mice in the C48/80-induced and SP-induced groups. The tail veins of these mice were then injected with 4% Evans Blue. Mice were anesthetized intraperitoneally with pentobarbital sodium (50 mg/kg) for 1 h, and the thickness of the sole on each hind paw was then measured. Next, the left hind paw was administered a 5 μL subcutaneous injection of C48/80 (30 μg/mL) or SP (4 μg/mL), while the right hind paw was administered a saline subcutaneous injection as a control. The thickness of each sole on the hind paws was subsequently measured again 15 min later. After euthanasia, the paws of the mice were dried at 50°C and weighed. The dried paws were then incubated in 500 μL of formamide for 8 h at 65°C to extract the Evans Blue. Finally, the supernatants were collected, and their optical density (OD) was measured at 630 nm.

### 2.5. Skin Avidin and Hematoxylin and Eosin (H&E) Stain Assay in C57BL/6 Mice

C57BL/6 mice were administered intraperitoneally with caffeine dissolved in 0.2 mL of physiological saline (0, 1, 2, 4 mg/kg) for 30 min. The pretreated mice were then anesthetized intraperitoneally with pentobarbital sodium (50 mg/kg). In the positive control group, 5 μL of 30 μg/mL C48/80 was subcutaneously injected into the footpad. In the negative control group, 5 μL of saline was subcutaneously injected into the footpad. After 15 min, the paw skin was excised and fixed in 4% formaldehyde for 48 h. The skin samples were then subjected to immunofluorescence staining (using FITC-avidin) and H&E staining. Images were subsequently captured using an inverted fluorescence microscope (Nikon, Ti-U, Japan).

### 2.6. Measurement of Body Temperature and Heart Rate in C57BL/6 Mice

C57BL/6 mice were successively injected with 0.2 mL of normal saline, normal saline, and caffeine (4 mg/kg) through the tail vein at 0 min. Twenty minutes later, 0.2 mL of normal saline, SP (4 μg/mL), and SP (4 μg/mL) were injected successively through the tail vein. Preheat the BP-2000 noninvasive blood pressure analysis system for small animals for 15 min before the formal experiment. After preheating, the mice were fixed on the blood pressure monitor. The mean arterial blood pressure and heart rate within 20 min after the first tail vein injection of 200 μL of the corresponding solution were measured. After the measurement was completed, the second tail vein injection was performed, and the mean arterial blood pressure and heart rate were measured for 20 min. Meanwhile, the body temperatures of each C57BL/6 mouse were measured once using the FT3403 thermometer at 5, 10, 15, and 20 min after the first injection, and at 5, 10, 15, and 20 min after the second injection, respectively.

### 2.7. Measurement of Serum Cytokines in C57BL/6 Mice

C57BL/6 mice were administered caffeine (0, 1, 2, 4 mg/kg) in 0.2 mL saline by injection into the tail vein. After 30 min, either C48/80 (0.2 mL, 30 μg/mL), SP (0.2 mL, 4 μg/mL), or saline (0.2 mL) was injected intravenously. After 1 h, blood supernatants were collected by centrifugation (12,000 rpm at 4°C for 20 min). Finally, TNF-α and MCP-1 levels were analyzed by ELISA.

### 2.8. Cytotoxicity and Apoptosis Assay

The toxicity of caffeine (0, 50, 100, 200, 400 μmol/L) to LAD2 cells was assessed using a CCK8 assay and an apoptosis kit. Apoptosis analysis was conducted using an AccuriC6 Plus flow cytometer. The potential of caffeine (50, 100, and 200 μmol/L) to activate β-hexosaminidase release and calcium influx in LAD2 cells was also investigated.

### 2.9. β-Hexosaminidase Assay

100 μL LAD2 cells (5 × 10^4^ cells/mL) were plated in 96-well plates. After removal of the medium, 50 μL of a modified bench solution [29] containing caffeine (0, 50, 100, 200 μmol/L) was added, and the cells were incubated for 30 min. C48/80 (30 μg/mL, 50 μL) or SP (4 μg/mL, 50 μL) was then added, and the cells were further incubated for 30 min. Next, the plates were centrifuged (1700 rpm, 5 min), and 50 μL of each supernatant was collected into new plates. The blank group cells were added 0.1% Triton X-100 (100 μL), then collected the supernatant (50 μL) after centrifuging for 5 min into new plates (as the lysate). The supernatant and the lysate in new plates were supplemented with 50 μL of β-hexosaminidase. Then incubated the plate at 37°C for 120 min. Finally, the termination solution (150 μL) was added, and OD_405 nm_ was measured by a microplate reader.

### 2.10. Chemokine Release Assay

96-well plates were seeded with 100 μL aliquots of LAD2 cells (5 × 10^4^ cells/mL) and cultured for 24 h. After removal of the medium, 150 μL of a modified bench solution [29] containing caffeine (0, 50, 100, 200 μmol/L) and either C48/80 (30 μg/mL) or SP (4 μg/mL) was added to each well. The plates were then incubated at 37°C in a 5% CO_2_ incubator for an additional 6 h. Finally, 100 μL of supernatant was collected from each well for measurement of MCP-1 and IL-8 levels using the relevant kit (according to manufacturer's instructions).

### 2.11. Intracellular Ca^2+^ Assay

96-well plates were seeded with either 100 μL LAD2 cells (5 × 10^4^ cells/mL) or 100 μL MRGPRX2-HEK293 cells (5 × 10^4^ cells/mL) and cultured for 24 h. The cells were then washed with calcium imaging buffer (CIB) [30] and resuspended in CIB containing caffeine (0, 50, 100, 200 μmol/L), 5 μmol/L Fluo-3 AM, and 0.1% F-127. The plates were then incubated in a dark incubator for an additional 30 min. Next, the cells were washed with CIB and subsequently resuspended in 50 μL CIB. The fluorescence was then monitored under a fluorescence microscope (Nikon, Tokyo, Japan) for 120 s. At 10 s after initial imaging, either C48/80 (30 μg/mL, 50 μL) or SP (4 μg/mL, 50 μL) was added.

### 2.12. Histamine Release Assay

96-well plates were seeded with either 100 μL LAD2 cells (5 × 10^4^ cells/mL) or 100 μL MRGPRX2-HEK293 cells (5 × 10^4^ cells/mL) and cultured for 24 h. The cells were then washed with modified bench solution, and 50 μL of modified bench solution containing caffeine (0, 50, 100, and 200 μmol/L) was added. The plates were then incubated at 37°C for an additional 30 min. Next, either C48/80 (30 μg/mL, 50 μL) or SP (4 μg/mL, 50 μL) was added, and the plates were again incubated for 30 min. Finally, each supernatant (50 μL) was collected, mixed with histamine internal standard (100 μL), and analyzed on an LC-MS-8040 (Shimadzu SSL).

### 2.13. siRNA Transfection of LAD2 Cells

We used a Siglec-6-targeted siRNA to achieve a specific knockout of Siglec-6 in LAD2 cells. SMART double-stranded siRNAs for Siglec-6 and a negative control (a nonspecific siRNA without a target) were synthesized by Shanghai GenePharma Co., Ltd. (Shanghai, China). The siRNA sequences were as follows:

Siglec-6 forward: 5′-CAGGCAUAGUUUCAGACCATT-3′.

Siglec-6 reverse: 5′-UGGUCGGAAACUAUGCCUGTT-3′.

Control forward: 5′-UUCUCCGAACGUGUCACGUTT-3′.

Control reverse: 5′-ACGUGACACGUUCGGAGAATT-3′.

LAD2 cells were transfected with siRNA-Siglec-6 (or the negative control) using Lipofectamine 3000 transfection reagent (20 nmol/L) from Invitrogen (Lot#: CN2481208). After 36 h of incubation, we used western blot analysis and RT-PCR to confirm the effectiveness of siRNA transfection. We noted that the specific expression of Siglec-6 was inhibited at the 36-h time-point. β-hexosaminidase release, histamine release, and Ca^2+^ mobilization were then investigated using the siRNA-Siglec-6-LAD2 cells (or LAD2 cells transfected with the negative control).

### 2.14. Western Blot

LAD2 cells, Siglec-6-siRNA LAD2 cells, and NC-siRNA LAD2 cells were treated with 30 μg/mL C48/80 or m-3M3FBS (20 μM) and caffeine (0, 25, 50, 100, 200 μmol/L) for 24 h. The cells were then harvested and lysed by RIPA (Epizyme Biotech, PC101) for subsequent western analyses. Total protein concentrations in the lysed samples were determined using the BCA method. Equal amounts of total protein were then subjected to electrophoresis on a 10% SDS–PAGE gel. The separated protein bands were then transferred to a membrane. After blocking, the membrane was incubated with the relevant primary and secondary antibodies prior to visualization. The primary antibodies used were as follows: anti-GAPDH (1:5000, 52,902) from Signalway Antibody; anti-MRGPRX2 (1:500) from lab-made; anti-SHP-1 (1:1000, # 108192-T40) from Sino-Biological; anti-phosphorylated-SHP-1 (p-SHP-1; 1:1000, #ab41436) from Abcam; anti-Siglec-6 (1:1000, ab317307) from Abcam; anti-PLCγ (1:1000, #5690) from CST; anti-phosphorylated PLCγ1 (p-PLCγ1; Ser1248) (1:1000, #8713) from CST; anti-IP3R (1:1000, #3763) from CST; anti-phosphorylated-IP3R (p-IP3R; Ser1756) (1:1000, #8548) from CST; anti-ERK1/2 (1:1000, 4695) from CST; and anti-phosphorylated ERK1/2 (p-ERK1/2; Thr202/Tyr204) (1:1000, 4370) from CST; Peroxidase AffiniPure Goat Anti-Rabbit lgG (H + L) (DY60202, 1:5000) from Deeyeebio; Peroxidase AffiniPure Goat Anti-Mouse lgG (H + L) (DY60203, 1:5000) from Deeyeebio; HRP-conjungated AffiniPure Goat Anti-Rabbit IgE (H + L) (sa00001−2, 1:10,000) from proteintech.

### 2.15. Molecular Docking

The three-dimensional structure of the MRGPRX2 protein (PDB ID: 7VV5) [31] was imported from the PDB protein database. Maestro 12.8 was then utilized to simulate the docking of the MRGPRX2 protein and caffeine. Next, the three-dimensional structure of the Siglec-6 protein (SMTL ID: 7aw6.1) was imported from the Swiss-model database. Maestro 12.8 was then utilized to simulate the docking of Siglec-6 protein and caffeine.

### 2.16. Surface Plasmon Resonance (SPR)

In order to analyze SPR, Siglec-6 (ACROBiosystems, SI6-H5256) protein (25 μg/mL) was immobilized on sensor chip CM5 (Cytiva Sweden AB, Uppsala, Sweden) by capture coupling. The interaction between Siglec-6 and caffeine was detected by Biacore T200 (General Electric Medical System, Fairfield, CT) at 25°C. The caffeine sample was prepared by 1xPBSP, and the mobile phase was 1xPBSP.

### 2.17. Statistical Analysis

All data are presented as mean ± S.D. Prior to statistical analysis, the normality of the data was assessed using the Shapiro–Wilk test. For the statistical comparison of means, we employed either analysis of variance (ANOVA) or an unpaired *t*-test. When the data were normally distributed and variances were equal (as assessed by Levene's test), ANOVA was conducted, followed by Tukey's multiple comparisons test. Conversely, when the data were not normally distributed or variances were unequal, an unpaired *t*-test with Welch's correction was utilized. Significance levels are denoted as *⁣*^*∗*^*p* < 0.05, *⁣*^*∗∗*^*p* < 0.01, and *⁣*^*∗∗∗*^*p* < 0.001. All statistical analyses were performed using GraphPad Prism 8.4.3 software.

## 3. Results

### 3.1. Caffeine Inhibits Secretagogue-Induced Local Anaphylactoid Reactions

**Figure 1:**
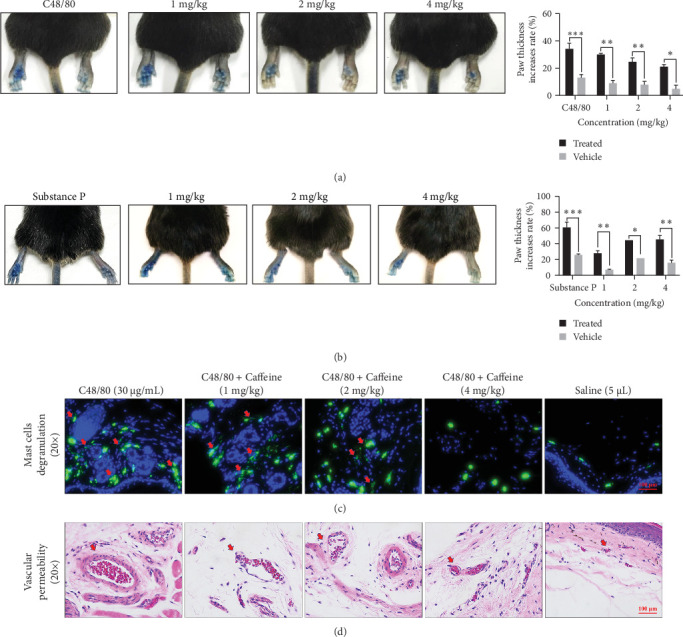
In vivo assessment the local anaphylactoid reaction of C57BL/6 mice treated with caffeine. (A) The exudation and thickness of hind paw (C48/80 mice model). (B) The exudation and thickness of hind paw (SP mice model). (C) The mast cells degranulation of mice hind paw skin tissue (avidin staining). (D) The vascular permeability of mice hind paw skin tissue (H&E staining), *n* = 2–3. Significance levels were denoted as *⁣*^*∗*^*p* < 0.05, *⁣*^*∗∗*^*p* < 0.01, and *⁣*^*∗∗∗*^*p* < 0.001.

Caffeine significantly reduced the back paw thickening observed in mice induced with C48/80 (Figure 1A) or SP (Figure 1B). Additionally, caffeine was observed to significantly reduce degranulation of MCs (Figure 1C) and capillary dilation (Figure 1D) in the skin. These results suggest that caffeine can alleviate local anaphylactoid reactions in mice after induction with C48/80 or SP.

### 3.2. Caffeine Inhibits Secretagogue-Induced Systemic Anaphylactoid Reactions

**Figure 2:**
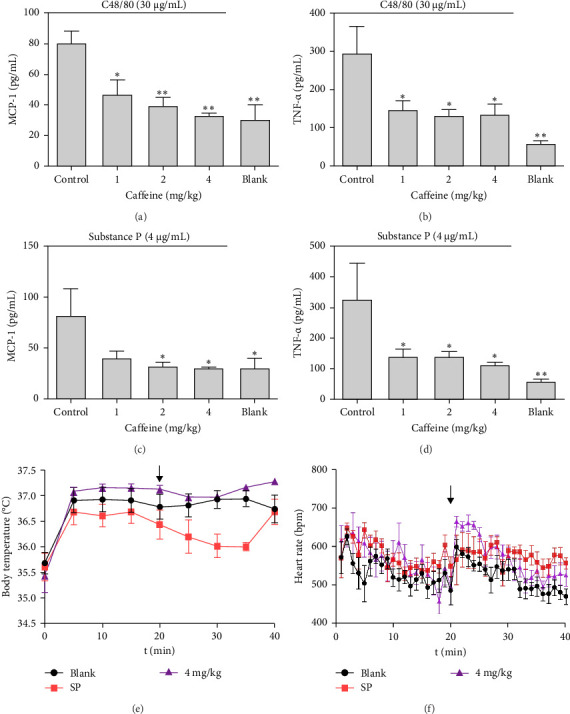
Serum factor release, body temperature and heart rate change of C57BL/6 mice treated with caffeine. (A, B) The serum MCP-1 and TNF-α release level in mice (caffeine cotreated with C48/80). (C, D) The serum MCP-1 and TNF-α release level in mice (caffeine cotreated with SP). (E) Body temperature change of mice. (F) Heart rate change of mice, *n* = 3–7. Significance levels were denoted as *⁣*^*∗*^*p* < 0.05, *⁣*^*∗∗*^*p* < 0.01, and *⁣*^*∗∗∗*^*p* < 0.001.

Caffeine pretreatment (at different concentrations) significantly reduced the spikes in serum concentrations of MCP-1 (Figure 2A,C) and TNF-α (Figure 2B,D) associated with induction by C48/80 or SP. Moreover, through experiments mouse model of systemic allergic reaction induced by SP. It was found that compared with the blank group, the body temperature of the model group dropped to the lowest value after about 15 min of treatment, and then there was a certain recovery. However, the body temperature of the caffeine treatment group showed no significant change compared with the model group (Figure 2E). The results indicated that caffeine had a better effect in alleviating the changes in body temperature during systemic allergic reactions. Compared with the blank group, the heart rate of the model group increased to a certain extent around 7 min of treatment, and its value tended to stabilize around 15 min. However, the caffeine treatment group was slightly better than the SP group in both the time and amplitude of stabilization (Figure 2F). This might be the reason why the measurement of body temperature indicators in mice is relatively sensitive. Blood pressure was also detected simultaneously, and results showed that a concentration of SP at 4 μg/mL did not cause changes in blood pressure due to systemic allergic reactions in mice. This might be because the blood pressure data is relatively stable, and the drug in this concentration model cannot cause changes in blood pressure. The above results suggest that caffeine can also alleviate systemic anaphylactoid reactions.

### 3.3. Caffeine Inhibits MRGPRX2-Induced MC Activation

Cell viability assays indicated that caffeine (0–400 μmol/L) had minimal toxic effects on LAD2 cells after 48 h of incubation (Figure S1A). Additionally, measurements of cell apoptosis indicated that long-term incubation with caffeine (0–400 μmol/L) had minimal (little to no) cytotoxic effects on LAD2 cells (Figure S1D). Caffeine also had no effect on β-hexosaminidase secretion (Figure S1B) or calcium influx (Figure S1C). Together, these findings provide evidence that caffeine has weak toxicity toward LAD2 cells.

**Figure 3:**
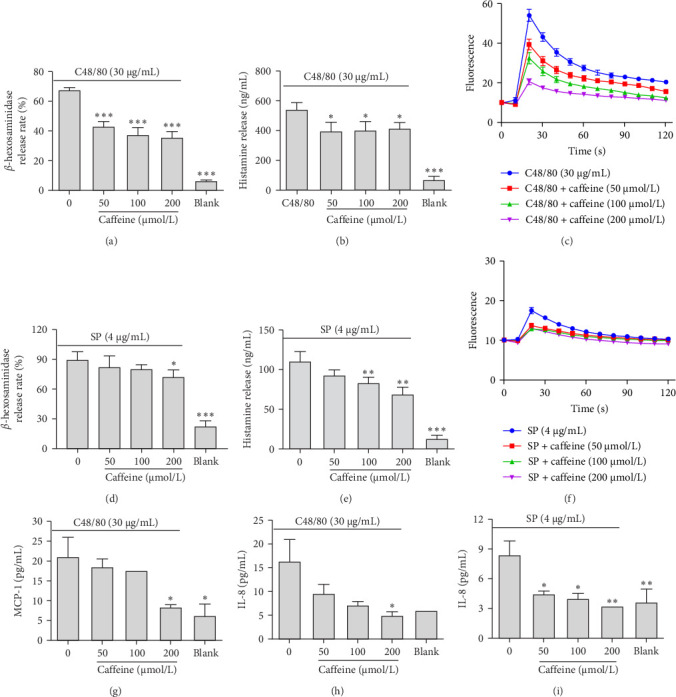
Caffeine attenuated LAD2 cells activation. (A–C) The release level of β-hexosaminidase, histamine, and Ca^2+^ (caffeine cotreated with C48/80). (D–F) The release level of β-hexosaminidase, histamine, and Ca^2+^ (caffeine cotreated with SP). (G, H) The release level of MCP-1 and IL-8 (caffeine cotreated with C48/80). (I) The release level of IL-8 (caffeine cotreated with SP), *n* = 2–3. Significance levels were denoted as *⁣*^*∗*^*p* < 0.05, *⁣*^*∗∗*^*p* < 0.01, and *⁣*^*∗∗∗*^*p* < 0.001.

Additionally, caffeine inhibited C48/80-triggered β-hexosaminidase secretion (Figure 3A), histamine secretion (Figure 3B), calcium flux (Figure 3C), MCP-1 secretion (Figure 3G), and IL-8 secretion (Figure 3H) in LAD2 cells. Caffeine also inhibited MC activation in SP-induced LAD2 cells, reducing β-hexosaminidase secretion (Figure 3D), histamine secretion (Figure 3E), calcium influx (Figure 3F), and IL-8 secretion (Figure 3I). Together, these results provide evidence that caffeine exhibits antianaphylactoid reaction activities.

**Figure 4:**
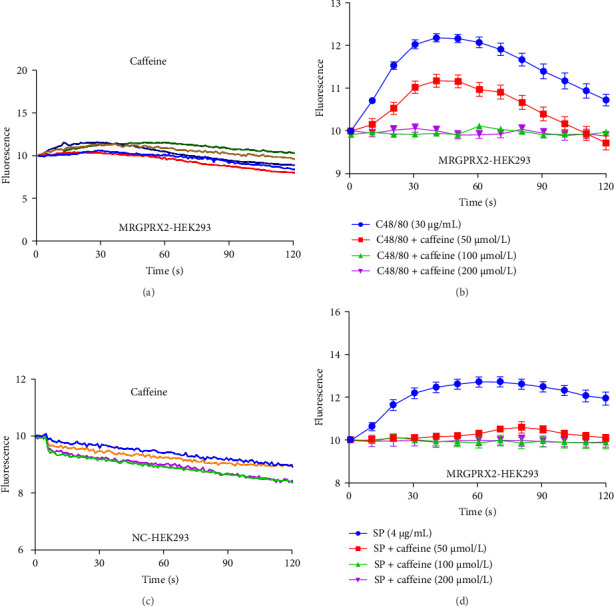
Caffeine inhibited Ca^2+^ release in MRGPRX2 high expression cells. (A) The level of Ca^2+^ release in MRGPRX2-HEK293 cells (200 μmol/L caffeine treated). (B) The level of Ca^2+^ release in MRGPRX2-HEK293 cells (caffeine cotreated C48/80). (C) The level of Ca^2+^ release in NC-HEK293 cells (200 μmol/L caffeine treated). (D) The level of Ca^2+^ release in MRGPRX2-HEK293 cells (caffeine co-treated with SP). NC-HEK293: HEK293 cells expressing empty plasmid, *n* = 3. Significance levels were denoted as *⁣*^*∗*^*p* < 0.05, *⁣*^*∗∗*^*p* < 0.01, and *⁣*^*∗∗∗*^*p* < 0.001.

In MRGPRX2-HEK293 cells, caffeine reduced C48/80-induced calcium influx (Figure 4B) and SP-induced calcium influx (Figure 4D). No excitatory effect was observed using caffeine only (Figure 4A,C). Hence, caffeine can regulate MRGPRX2-induced MC activation.

### 3.4. Caffeine Inhibits MRGPRX2-Induced MC Degranulation via Activation of Siglec-6

**Figure 5:**
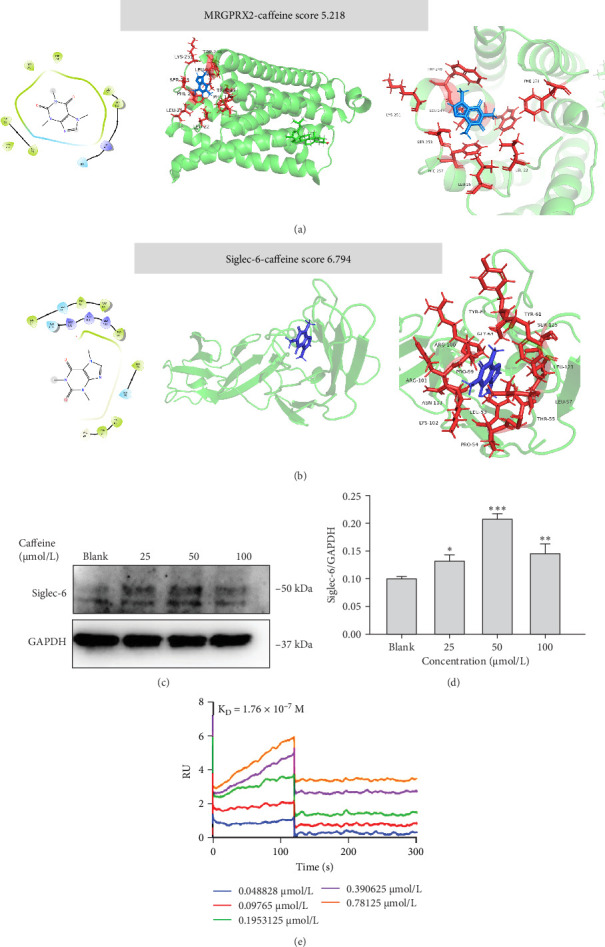
Caffeine binding characteristics on Siglec-6 receptor. (A) The interactions between caffeine with MRGPRX2, (B) the interactions between caffeine with Siglec-6, (C) the Siglec-6 protein level of caffeine treated LAD2 mast cell, (D) quantitative chart of Siglec-6 expression, and (E) the interaction between caffeine and Siglec-6 by SPR, *n* = 3. Significance levels were denoted as *⁣*^*∗*^*p* < 0.05, *⁣*^*∗∗*^*p* < 0.01, and *⁣*^*∗∗∗*^*p* < 0.001.

**Figure 6:**
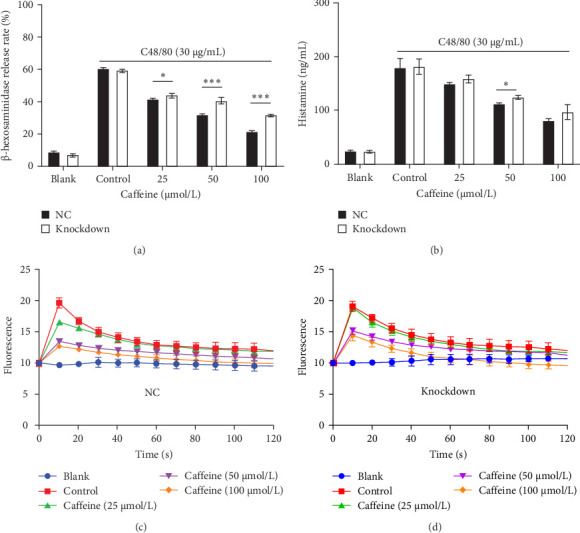
Mast cells were activated after caffeine intervention in siRNA-Siglec-6-LAD2 cells. (A, B) The level of β-hexosaminidase and histamine in NC-LAD2 cells, and siRNA-Siglec-6-LAD2 cells. (C) The Ca^2+^ level of NC-LAD2 cells. (D) The Ca^2+^ level of siRNA-Siglec-6-LAD2 cells, *n* = 3. Significance levels were denoted as *⁣*^*∗*^*p* < 0.05, *⁣*^*∗∗*^*p* < 0.01, and *⁣*^*∗∗∗*^*p* < 0.001.

Potential ligand-protein interactions between caffeine and MRGPRX2 were investigated by molecular docking. The docking results with MRGPRX2 revealed that the ligand established hydrophobic contacts with multiple amino acid residues, including LEU22, LEU25, LEU247, PHE170, PHE257, TRP243, TRP248, LYS251, and SER253. Among these, aromatic residues such as PHE170, TRP243, and TRP248 were proximally distributed to the aromatic ring of the ligand, suggesting potential stacking interactions (Table 1). The docking score was 5.218, exhibiting a weak binding interaction with MRGPRX2 (Figure 5A, Table 1). We next investigated potential ligand-protein interactions between caffeine and Siglec-6 using molecular docking. The docking results with Siglec-6 showed that the caffeine molecule was primarily surrounded by residues such as LEU57, PHE58, ASP203, ASN202, PHE64, LEU85, ARG100, LEU123, TRP46, and GLY43, involving hydrophobic contacts, aromatic ring stacking, and spatial fitting. The aromatic side chains of PHE64 and PHE58 were adjacent to the planar structure of the ligand, creating steric hindrance that facilitated ligand positioning. Meanwhile, ASP203 and ASN202 provided a polar interaction environment, though no significant hydrogen bonding was observed (Table 1). The docking score was 6.794, exhibiting a stronger interaction with Siglec-6 (Figure 5B, Table 1). Using western blot experiments, we subsequently confirmed that caffeine could upregulate the expression of Siglec-6 protein (Figure 5C,D). SPR assay was performed to directly validate the interaction between caffeine and Siglec-6. The binding affinity between caffeine and Siglec-6 protein is with a calculated *K*_D_ of 1.76 × 10^−7^ mol/L (Figure 5E). The data demonstrated a direct and specific interaction between caffeine and Siglec-6. Next, we used western blot and RT-PCR analyses to verify the knockdown effects of Siglec-6 siRNAs. The results shown in Figure S2 demonstrate that the 1231 sequence of Siglec-6 had the best knockdown effect. Therefore, the 1231 sequence was selected for all subsequent knockdown experiments. siRNA-Siglec-6-LAD2 cells exhibited similar C48/80-induced β-hexosaminidase secretion (Figure 6A), histamine secretion (Figure 6B), and calcium influx (Figure 6D) to NC-LAD2 cells (Figure 6A–C). However, β-hexosaminidase secretion, histamine secretion, and calcium influx were significantly increased in siRNA-Siglec-6-LAD2 cells after caffeine pretreatment (compared with NC-LAD2 cells) (Figure 6A–D). Together, the above results provide evidence that downregulation of Siglec-6 expression attenuates the ability of caffeine to inhibit activation of MCs.

### 3.5. Caffeine Inhibits MRGPRX2 Signal Transduction via the Siglec-6/SHP-1-PLC-γ1/IP3R-Ca^2+^ Pathway

**Figure 7:**
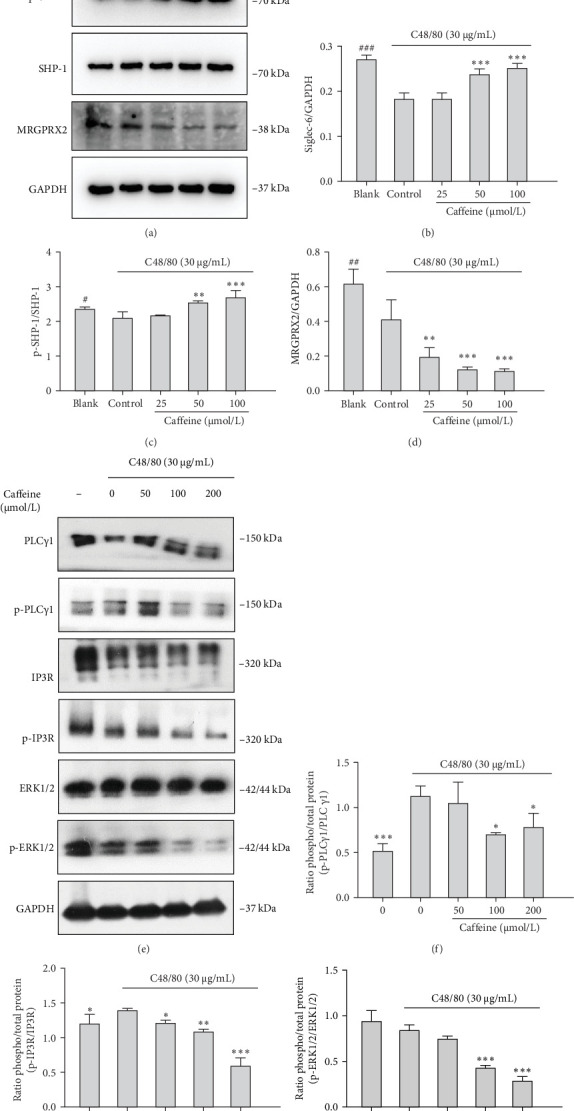
Caffeine upregulation Siglec-6/SHP-1 signaling pathway and downregulation MRGPRX2 signaling pathway. (A) The levels of Siglec-6, p-SHP-1, SHP-1, and MRGPRX2 protein. (B–D) Quantification of Siglec-6, p-SHP-1, and MRGPRX2 protein expression. (E) The levels of PLCγ1, p-PLCγ1, IP3R, p-IP3R, ERK1/2, and p-ERK1/2 by western blot. (F–H) Quantification of p-PLC, p-IP3R, and p-ERK1/2 expression, *n* = 3. Significance levels were denoted as *⁣*^*∗*^*p* < 0.05, *⁣*^*∗∗*^*p* < 0.01, and *⁣*^*∗∗∗*^*p* < 0.001.

Caffeine upregulated expression levels of Siglec-6 (Figure 7A,B) and p-SHP-1 (Figure 7A,C), while reducing MRGPRX2 (Figure 7A,D) and the phosphorylation levels of downstream signaling proteins such as p-PLC-γ1, p-IP3R, and p-ERK1/2 (Figure 7E–H). In summary, modulation of Siglec-6 by caffeine negatively regulated the activation of MCs by phosphorylating SHP-1 and dephosphorylating signaling proteins downstream of MRGPRX2.

### 3.6. Siglec-6 is Required for Caffeine-Mediated SHP-1 Activation and MRGPRX2 Suppression

**Figure 8:**
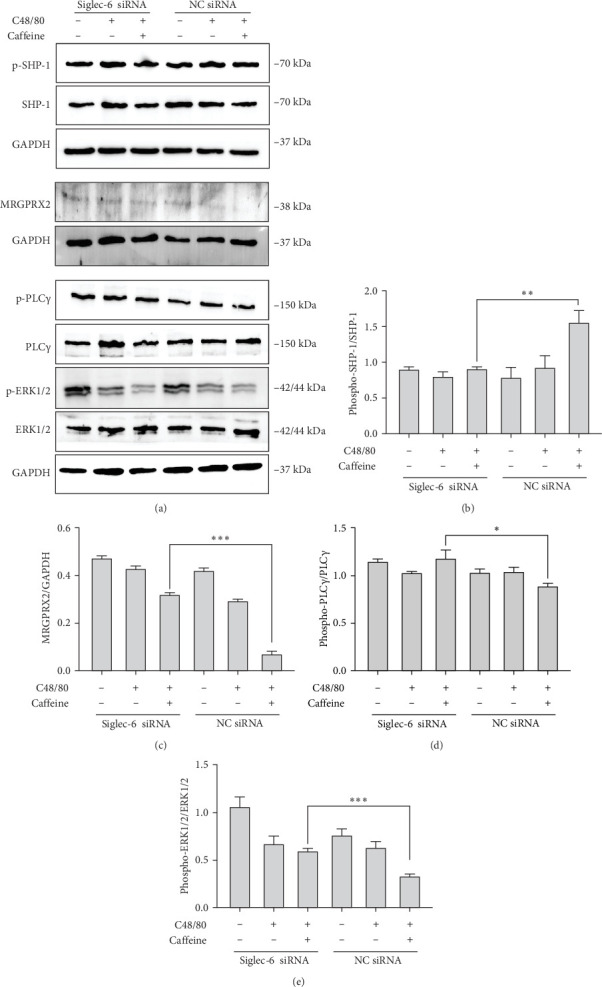
Caffeine targeted Siglec-6 downregulation MRGPRX2 signaling pathway. (A) The levels of p-SHP-1, SHP-1, MRGPRX2, PLCγ1, p-PLCγ1, ERK1/2, and p-ERK1/2 protein in Siglec-6 siRNA-LAD2 cells. (B) Quantification of p-SHP-1 protein expression. (C) Quantification of MRGPRX2 protein expression. (D) Quantification of p-PLCγ1 expression. (E) Quantification of p-ERK1/2 expression, *n* = 3. Significance levels were denoted as *⁣*^*∗*^*p* < 0.05, *⁣*^*∗∗*^*p* < 0.01, and *⁣*^*∗∗∗*^*p* < 0.001.

To verify the role of Siglec-6 in mediating caffeine's effects on SHP-1 and MRGPRX2, interference of Siglec-6 expression in LAD2 MCs was performed. Western blot demonstrated that phosphorylation of SHP-1 showed no increase with caffeine treatment in Siglec-6-knockdown cells than in NC-knockdown cells (Figure 8A,B). Caffeine's downregulation of MRGPRX2 (Figure 8A,C), p-PLCγ (Figure 8A,D), and p-ERK1/2 (Figure 8A,E) was abolished in Siglec-6-knockdown cells than in NC-knockdown cells. These results suggested that caffeine upregulates Siglec-6 expression and downregulates MRGPRX2. Siglec-6 is required for caffeine-mediated SHP-1 activation and MRGPRX2 suppression.

**Figure 9:**
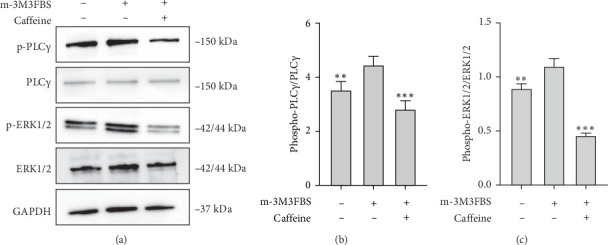
Caffeine inhibits PLC downstream signaling pathways. (A) The levels of PLCγ1, p-PLCγ1, ERK1/2, and p-ERK1/2 in m-3M3FBS-activated LAD2 cells. (B) Quantification of p-PLCγ1 expression. (C) Quantification of p-ERK1/2 expression, *n* = 3. Significance levels were denoted as *⁣*^*∗*^*p* < 0.05, *⁣*^*∗∗*^*p* < 0.01, and *⁣*^*∗∗∗*^*p* < 0.001.

To observe the changes in the downstream signaling pathways of PLC, the reverse of PLC expression operation was conducted; we pretreated LAD2 cells with the PLC agonist m-3M3FBS (20 μM). As shown in Figure 9, the PLC agonist m-3M3FBS activates downstream signaling molecules of PLC, including p-PLCγ1 and p-ERK1/2, whereas caffeine suppressed the m-3M3FBS-induced upregulation of p-PLCγ1 and p-ERK1/2 levels. This demonstrated that caffeine inhibits the MRGPRX2-PLCγ1-ERK1/2 signaling pathway.

## 4. Discussion

In this study, we found that caffeine inhibited local and systemic anaphylactoid reactions and MC degranulation. Although caffeine suppressed anaphylactoid reactions by inhibiting MRGPRX2-induced degranulation in MCs, it does not directly bind MRGPRX2. Instead, caffeine stimulated Siglec-6 expression and activation, triggering a negative feedback mechanism to inhibit MRGPRX2 signal transduction. Western blot experiments demonstrated that caffeine negatively regulated MRGPRX2-induced anaphylactoid reactions through the Siglec-6/SHP-1-PLC-γ1/IP3R-Ca^2+^ molecular pathway. Taken together, the above results provide an indication for the application of caffeine in anaphylactoid reactions and disease treatment.

An anaphylactoid reaction is a clinical syndrome with a rapid onset that is induced by multiple factors, caused by different immune or nonimmune mechanisms, and involves multiple organs. In clinics, there have been reports of skin flare-ups, rashes, and even severe shock reactions after administering certain drugs. Most of the therapeutic drugs used for the treatment of allergic diseases are antihistamines, which alleviate the symptoms of patients (to some extent) but cannot completely cure them. Some patients may also develop drug resistance, so there is an urgent need for new candidate drugs.

Caffeine is found in everyday beverages such as coffee, tea, and energy drinks [32]. Previous studies have found that caffeine alleviates some of the symptoms of allergic diseases such as bronchial asthma and allergic reactions [19, 20]. However, the mechanism underlying caffeine inhibition of anaphylactoid reactions remains unclear. Here, for the first time, we demonstrate that caffeine can inhibit anaphylactoid reactions in mice, reducing MC degranulation and cytokine release. Previous studies have only tentatively revealed the antianaphylactoid effects of caffeine.

Further experiments revealed that caffeine reduced SP-induced and C48/80-induced calcium influx in MRGPRX2-HEK293 cells, suggesting that caffeine may regulate MRGPRX2-induced activation of MCs. MRGPRX2 has emerged as a promising target in the development of new therapies for allergic diseases. MRGPRX2 is widely expressed on human dendritic cells, basophils, and MCs, and it is known to be an important target in anaphylactoid reactions [21]. The cryo-electron microscopy (cryo-EM) structure of the MRGPRX2-Gαi trimer in complex with C48/80 or inflammatory peptides has been reported, and the consensus sequence of peptide allergens has been mapped. These findings have laid a solid structural foundation for drug discovery efforts targeting MRGPRX2 [31, 33]. However, based on our molecular docking results, the interaction between caffeine and MRGPRX2 (score: 5.218) is relatively weak. These results suggested that caffeine does not affect inhibition of anaphylactoid reactions via MRGPRX2, and further research on the mechanism of caffeine is needed.

MCs play important roles in innate and adaptive immunity [34, 35]. Siglecs are also known to play a role in the treatment of allergic diseases and inflammatory responses. Members of the Siglec family regulate the activation and signal transduction of immune cells by interacting with salivary glycan chains, thereby influencing the occurrence and progression of allergic reactions [9]. Specifically, Siglec-6 reduces the release of inflammatory mediators by inhibiting MC activation and regulating B cell function [10, 11]. Siglec-8 alleviates allergic inflammation by inducing eosinophil apoptosis and inhibiting MC activation [10]. Siglec-9 reduces inflammatory responses by inhibiting the activation of monocytes and neutrophils. Siglec-10 promotes immune tolerance, thereby reducing the incidence of allergic reactions. MCs have been shown to express Siglec receptors with ITIMs that help suppress their activation. Siglec receptors with ITIMs recruit protein kinases to phosphorylate signaling molecules and inhibit tyrosine phosphatases (PTPs) such as SHP-1/2 [26, 36–39]. Siglec-6 is highly expressed in MCs, and it encodes an ITIM [26, 40, 41]. Because of its high selectivity, stable expression, and strong inhibitory capacity, Siglec-6 is a promising therapeutic target for intervention in MC-based allergic diseases [27]. Here, we report that caffeine exhibits a strong interaction with Siglec-6. Moreover, caffeine was shown to upregulate the expression of Siglec-6, providing evidence that caffeine promotes Siglec-6 function and ultimately inhibits the activation of MCs. The binding affinity between caffeine and Siglec-6 protein is with a calculated *K*_D_ of 1.76 × 10^−7^ mol/L. The data demonstrated a direct and specific interaction between caffeine and Siglec-6. Although our research mainly focuses on Siglec-6, other members of the Siglec family may also interact with caffeine. Therefore, in our future research, we will further explore the interaction between other members of the Siglec family and caffeine. The research presented here provided a new perspective on the role of Siglec-6 in allergic reactions, and it offers a new therapeutic target for allergic reactions. However, the mechanism underlying this process needs further clarification.

Siglec-6 interactions significantly weaken the activation of MCs through MRGPRX2 [27], most probably by regulating intracellular signaling by phosphorylation of SHP-1 and SHP-2 [28]. Our working hypothesis was that caffeine activates Siglec-6 function, which triggers a negative feedback mechanism to inhibit MRGPRX2-dependent degranulation of MCs. Additional experiments confirmed that caffeine upregulated SHP-1 phosphorylation levels and downregulated levels of MRGPRX2 and its downstream signaling pathways, including the phosphorylation levels of PLC-γ1, IP3R, and ERK1/2. Western blot demonstrated that p-SHP-1 protein showed no increase with caffeine treatment in Siglec-6-knockdown cells than in NC-knockdown cells. The MRGPRX2, p-PLCγ1, and p-ERK1/2 treated with caffeine were abolished in Siglec-6-knockdown cells than in NC-knockdown cells. This demonstrated that Siglec-6 is required for caffeine-mediated SHP-1 activation and MRGPRX2 suppression.

The PLC agonist m-3M3FBS activated downstream signaling molecules of MRGPRX2, including p-PLCγ1 and p-ERK1/2, whereas caffeine suppressed the m-3M3FBS-induced upregulation of p-PLCγ1 and p-ERK1/2 levels. This demonstrated that caffeine inhibits the MRGPRX2-PLCγ1-ERK1/2 signaling pathway, confirming PLC's essential role.

Together, our results provide evidence that caffeine modulates Siglec-6 function, which negatively regulates the activation of MCs by phosphorylating SHP-1 and dephosphorylating downstream signaling proteins of MRGPRX2, ultimately inhibiting local and systemic anaphylactoid reactions.

## 5. Conclusion

Our results demonstrated that caffeine inhibits local and systemic anaphylactoid reactions induced in mice, and they provide evidence that caffeine triggers a Siglec-6-dependent feedback mechanism that eventually inhibits MRGPRX2-dependent degranulation of MCs. The molecular mechanism underlying this process includes the Siglec-6/SHP-1-PLC-γ1/IP3R-Ca^2+^ pathway.

## Figures and Tables

**Table 1:** Detailed data on molecular docking binding forces.

Molecular docking parameters	Caffeine-MRGPRX2	Caffeine-Siglec-6
Docking score	5.218	6.794
Key binding residue	LEU22, LEU25, LEU247, PHE170, PHE257, TRP243, TRP248, LYS251, SER253	LEU57, PHE58, ASP203, ASN202, PHE64, LEU85, ARG100, LEU123, TRP46, GLY43
Interaction type	Hydrophobic contact, aromatic ring stacking, hydrophobic effect	Hydrophobic contact, aromatic stacking, spatial fitting
Aromatic stacking	PHE170, TRP243, TRP248	PHE64, PHE58
Polar residue interaction	No obvious hydrogen bonds were formed	ASP203 and ASN202 provide a polar interaction environment
Ligand localization characteristics	Aromatic stacking	PHE64 and PHE58 provide steric hindrance, restricting ligand positioning

## Data Availability

The research data are not shared.

## Nomenclature

GPCRs: G-protein-coupled receptors
MRGPRX2: Mas-related G protein-coupled receptor X2
MCs: Mast cells
NMBDs: Neuromuscular blocking drugs
C48/80: Compound 48/80
SP: Substance P
C5aR: Complement component 5a receptor
CIB: Calcium imaging buffer
ITIMs: Immunoreceptor tyrosine-based inhibition motifs
ITAMs: Immunoreceptor tyrosine-based activation motifs
ITSM: Immunoreceptor tyrosine-based switch motif
SPR: Surface plasmon resonance.

## Supporting Information

Supporting Information Figure S1. Variability of caffeine on LAD2. Figure S2. The validation for the silenced gene sequence of Siglec-6.
