# Supplementary material for: Caffeine Acts as an Agonist of Siglec-6, Inhibits MRGPRX2-Triggered Mast Cell Degranulation and Anaphylactoid Reactions
Source: Mediators Inflamm. 2025 Aug 30;2025:9580121. doi: 10.1155/mi/9580121 (PMC12413947; doi:10.1155/mi/9580121)
Supplement: Supporting Information — Figure S1. Variability of caffeine on LAD2. Figure S2. The validation for the silenced gene sequence of Siglec-6. [file 9580121.f1.pptx]

## Slide 1
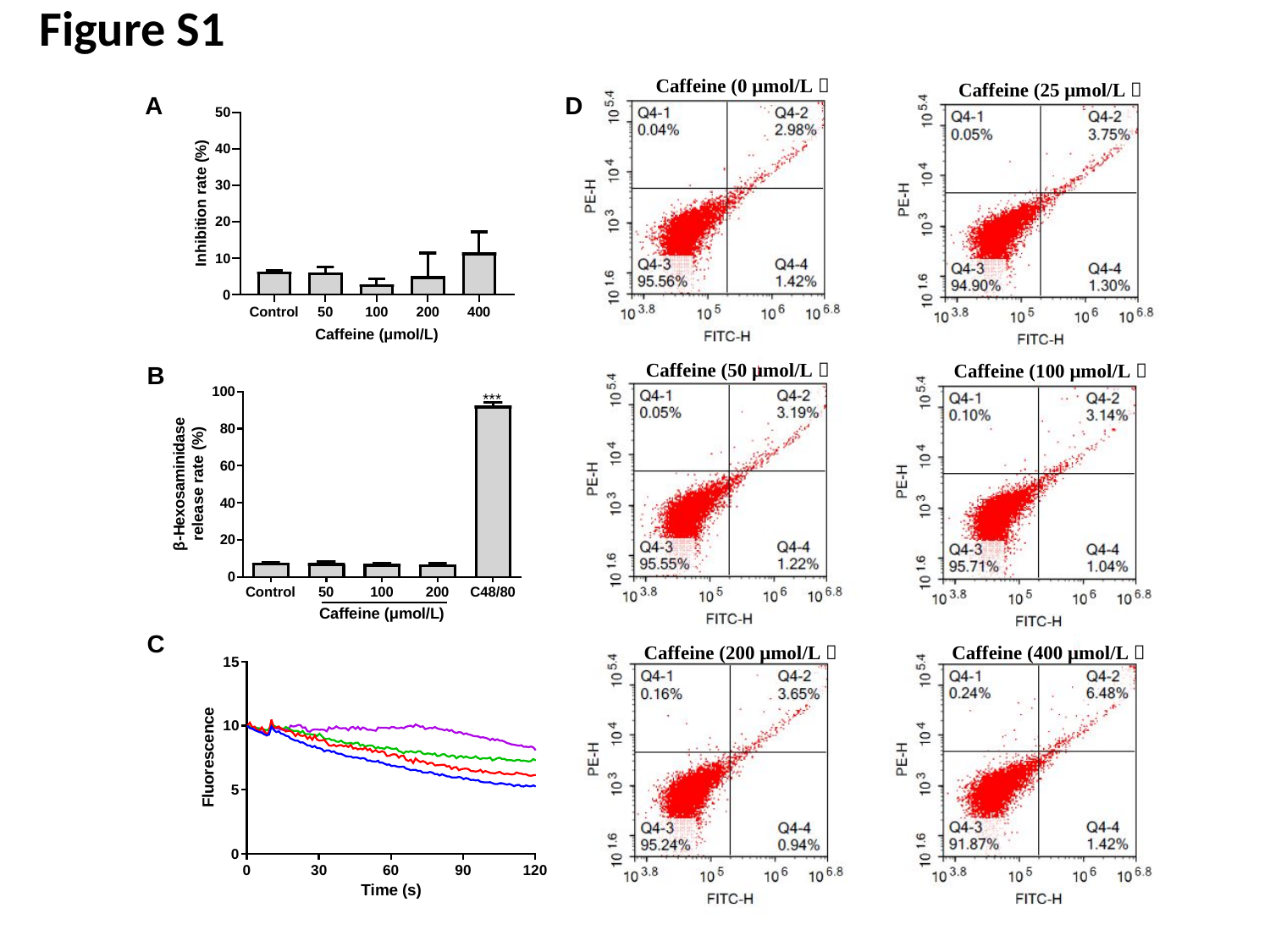

Figure S1
Caffeine (0 μmol/L）
Caffeine (25 μmol/L）
A
D
Caffeine (50 μmol/L）
Caffeine (100 μmol/L）
B
C
Caffeine (200 μmol/L）
Caffeine (400 μmol/L）

## Slide 2
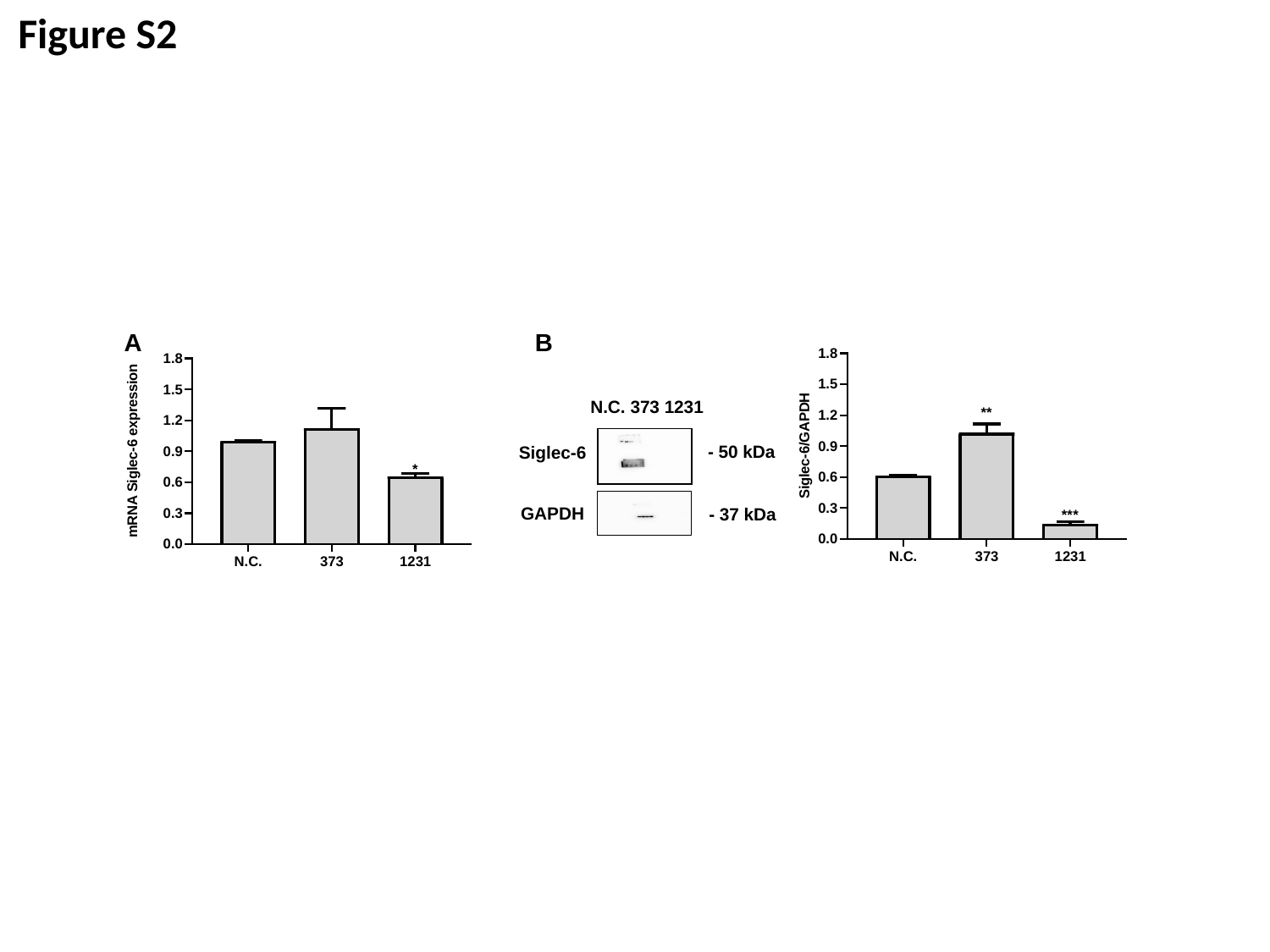

Figure S2
A
B
N.C. 373 1231
- 50 kDa
Siglec-6
GAPDH
- 37 kDa
